# Some Account of a New Extract of Bark Prepared in South America

**Published:** 1790

**Authors:** William Saunders

**Affiliations:** Physician to Guy's Hospital


VIII. *Some Account of a new Extract of Bark prepared in South America. Communicated in a Letter to Dr.* Simmons *by* William Saunders, *M. D. Phyfician to Guy's Hofpital.*

*To Dr.* Simmons.

Dear Sir,

AGREEABLY to your requeft, I now fend you the following particulars relative to the new extract of Peruvian bark prepared in

South America, and lately imported into this country from Spain as an article of commerce.

It is of a confiftence between the foft and hard extracts of the fhops; of a dark colour, and beautifully tranfparent. It is extremely foluble in the mouth, and has none of that empyreumatic or burnt tafte, fo common to all extracts, and which obfcures their original powers fo much as to have brought them into general difcredit. It has the tafte and flavour of the beft Peruvian bark in a very concentrated form. It is very foluble in boiling water, and when gently agitated with it, in the proportion of two drachms to a pint of water, it gives an impregnation more powerful than that of a decoction of bark in the proportion of an ounce of bark to a pint of water, prepared agreeably to the ufual formula for that purpofe.

It is more difficultly foluble in cold water.

One ounce of it foftened with two ounces of boiling water, and digefted with one quart of proof fpirit, in a gentle heat, gives a more powerful tincture than that of the Difpenfatory; the refiduum left on the filter weighs two fcruples, and is perfectly infipid.

It differs very materially from all other extracts tracts of bark with which it has been compared; and even from some which was carefully prepared from the beft bark, and flowly evaporated in a water bath. In its union with boiling water it refembles fo much the decoction of the pale bark, both in colour and fenfible qualities, that the difference is not perceptible; and by this fynthetic teft it may be diftinguifhed from all other extracts of bark.

In collecting, from various druggifts, extracts, with a view to a comparifon, many of them evidently appeared to be fophifticated by being chiefly compofed of the extract of gentian, an article of the materia medica better formed for that procefs than almoft any other.

No information has been received relative to the method of preparing this extract in South America; we are therefore left to conjecture that it may have the advantage of an aqueous folution from recent vegetable matter, and that the infpiffation or evaporation is conducted by an expofure to air and the heat of the fun.

All who have feen it admit its fuperior elegance, and that it poffeffes the fenfible qualities of the beft bark in the moft foluble and concentrated form. I have made frequent trials of it both in the hofpital and in private practice, and have have uniformly found that it has done every thing which could be expected from the beſt Peruvian bark in any form. I have had the ſame favourable report of its operation from other practitioners.

It ſits eaſy on the ſtomach, and in caſes of great emergence, as in gangrene and malignant fevers, or the putrid diſeaſes of warm climates, where the life of a patient may depend on the quantity of efficacious bark taken in a few hours, it muſt have a decided advantage. A patient may take four ounces of this extract in a day, a quantity equal in power and effect to a pound and a half of the beſt bark.

It is found efficacious in the cure of fevers, in the form of a clyſter; for which purpoſe I have diſſolved a drachm of it in four ounces of water. This method of preſcribing it is well adapted to children, and to ſuch patients as cannot retain bark in any form on the ſtomach.

The quantity at preſent in this country, I am informed, is all that has been introduced into Europe, and unleſs frauds are committed, and it becomes the ſubject of adulteration, it promiſes to become a very important acquiſition to the liſt of our uſeful and active remedies.

The ſolution of it in boiling water will be found found a ready and eafy fubftitute for the decoction of bark, and at an expence not exceeding the decoction of fuch bark as ought generally to be employed.

I am, Dear Sir,
Yours fincerely,
William Saunders.

*New Broad Street,*

February 11th, 1790.

